# Evaluation of Nrf2/Keap1 Pathway in Patients with Migraine

**DOI:** 10.3390/medicina61101732

**Published:** 2025-09-24

**Authors:** Fatih Koçtürk, Firdevs Emekli, Kadir Eği, Seyithan Taysi

**Affiliations:** 1 Department of Neurology, Faculty of Medicine, Kahramanmaras Sutcu Imam University, 46040 Kahramanmaras, Turkey; drfatihkocturk@gmail.com; 2 Department of Medical Biochemistry, Faculty of Medicine, Gaziantep University, 27310 Gaziantep, Turkey; dytfirdevsemekli@gmail.com

**Keywords:** migraine, Nrf2, Keap1, oxLDL, TAS, TOS

## Abstract

*Background and Objectives*: Migraine is the most common primary headache disorder worldwide, negatively affecting quality of life and limiting the functionality of individuals. Although its pathogenesis is not fully understood, it is known that activation of the trigeminovascular system, neurogenic inflammation, and oxidative stress are among the main components of migraine. In this context, we aimed to investigate the possible role of the nuclear factor erythroid 2-related factor 2 (Nrf2)/Kelch-like ECH-associated protein 1 (Keap1) signaling pathway, which plays a key role in the regulation of cellular oxidative stress, in the development of chronic diseases such as migraine. *Materials and Methods*: In this study, the oxidative stress parameters total oxidant level (TOS), total antioxidant level (TAS), and oxidative stress index (OSI) and changes in the Nrf2/Keap1 signaling pathway were analyzed in migraine patients. *Results*: The results showed that Keap1 levels were significantly higher in migraine patients compared with the control group, whereas the Nrf2 and TAS levels were low. In addition, increased levels of oxidized LDL (oxLDL) and glycogen synthase kinase-3 beta (GSK3B), which are oxidative stress markers, confirmed that the oxidative stress burden was high in migraine patients. The fact that OSI values were significantly higher in migraine patients clearly demonstrates that systemic oxidative stress was out of balance in these individuals. *Conclusions*: In conclusion, this study reveals that oxidative stress and the Nrf2/Keap1 signaling pathway play an important role in the pathogenesis of migraine. Decreased Nrf2 activity and increased Keap1 levels suggest that the antioxidant defense system is insufficient in migraine patients. These findings suggest that the Nrf2/Keap1 signaling pathway may be considered as a potential target for migraine treatment and that the development of new treatment strategies to reduce oxidative stress may be beneficial.

## 1. Introduction

Migraine is a disabling primary headache disorder that directly affects more than one billion people worldwide [1]. Migraine is one of the most common primary headache disorders with an annual prevalence of nearly 15% worldwide and is significantly disabling [2,3]. According to the Global Burden of Disease Study, migraine is the second most common neurological disorder worldwide, causing more disability than all other neurological disorders combined [3,4]. Migraine is characterized by symptoms such as vomiting, problems with vision, hearing and smell perception, and headaches lasting for at least 15 days per month. In addition, migraine often occurs together with other health problems, such as sleep problems, anxiety, and depression. This situation worsens the general health status of patients and negatively affects their quality of life [5]. Migraine is usually characterized by recurrent headache attacks and these attacks are often accompanied by different symptoms [6]. In approximately one-third of migraine patients, headaches are occasionally or constantly accompanied by or preceded by transient neurologic disorders known as migraine aura [7]. Moreover, in some individuals, the frequency of migraine attacks may increase and develop into a condition called chronic migraine [8]. Chronic migraine not only causes physical and difficulties for individuals, but also creates a significant burden for society [9]. It is widely accepted that both central and peripheral activation of the trigeminovascular system plays a role in the pathogenesis of migraine [10]. Furthermore, the neurophysiologic mechanism underlying migraine aura is thought to be cortical spreading depression [11].

Migraine is a complex and multidimensional neurologic disease that can usually persist for several days [12]. Pain, the main symptom of migraine, may not always be the most disturbing symptom for all patients [13,14]. The disease is usually characterized by four main interrelated phases: prodromal, aura, pain, and postdromal phases [12,15,16]. A better understanding of these processes has led to the consideration of migraine as a network disorder involving many cortical, subcortical and brainstem regions, producing a variety of signs and symptoms [17]. The precursor phase of migraine may start up to 3 days before the headache and some patients may anticipate that the headache will start in approximately 12 h [15]. Symptoms such as fatigue, mood changes, food cravings, yawning, muscle tenderness, and photophobia, which are frequently observed in this phase, suggest that the hypothalamus, brainstem, limbic system, and certain cortical areas are involved in the process [18,19,20]. Furthermore, migraine attacks may show a daily rhythm and are often triggered by changes in homeostasis [21]. These data suggest that chronobiologic mechanisms are involved in the pathogenesis of migraine and the importance of investigating the hypothalamus as a possible starting point [19,21].

The trigeminovascular system consists of the peripheral axons of the trigeminal ganglion that peripherally innervate the meninges and intracranial blood vessels. This structure merges centrally with the spinal trigeminal nucleus caudalis called the trigeminocervical complex (TCC) and the upper cervical spinal cord [22]. Second-order neurons arising from the TCC ascend to thalamocortical neurons and also project to basic nuclei in the diencephalon and brainstem, including the locus coeruleus (LC), periaqueductal gray (PAG), and hypothalamus [23,24]. The activation of trigeminovascular pain pathways regulates certain features of migraine pain through the release of neuropeptides such as calcitonin gene-related peptide (CGRP) and pituitary adenylate cyclase-activating polypeptide (PACAP) at the dura mater level [25,26,27]. CGRP is widely present in both peripheral and central neurons and has a strong vasodilatory effect. It also plays a modulatory role in central pain mechanisms by exerting regulatory effects on second- and third-order neurons [25]. The triggering of migraine pain begins with the stimulation of nociceptive neurons innervating the dura mater. In this process, vasoactive neuropeptides such as calcitonin gene-related peptide (CGRP) and pituitary adenylate cyclase-activating polypeptide-38 are released, and this leads to signal transduction along the trigeminovascular pathway. However, the extent to which arterial vasodilation, mast cell degranulation, and plasma extravasation contribute to this process has not yet been fully clarified [28,29].

Cranial autonomic symptoms such as lacrimation, nasal congestion and rhinorrhea together with symptoms such as nausea, vomiting and thirst indicate changes in autonomic functions in the central nervous system during migraine [30,31]. In this context, it has been shown that changes in sympathetic and parasympathetic tone can last from the premonitory phase to the postdromal phase [32]. According to one theory, migraine triggers such as stress, awakening, or other changes in physiological or emotional homeostasis may activate nociceptive pathways through increased parasympathetic tone [33]. The mechanisms of onset of a migraine attack are not fully understood. Although many patients identify extrinsic factors such as bright light, loud noises, and certain foods as triggers, experimental evidence supporting the effect of such triggers is limited [34,35]. In addition, a subjective reduction in stress perceived by patients has been found to be more strongly associated with an acute migraine attack. However, many patients report that the frequency of headaches increases when they are stressed [16]. Stimulating symptoms in migraine have a wide range and can be classified as homeostatic or hormonal changes, mood and fatigue, migraine symptoms, sensory sensitivities, and cranial autonomic symptoms [18]. Various studies in patients of different age groups have shown that yawning is a common and reliable symptom, while fatigue and mood changes are also frequently observed [16].

The biological structure of migraine is a highly complex process involving multiple factors, some aspects of which are still poorly understood. It is thought that genetic predisposition, and behavioral and environmental factors combine to cause changes in sensory brain function. These changes lead to increased sensory sensitivity, causing normal sensory stimuli to be perceived uncomfortably by migraine patients [12]. Moreover, the initial stage of migraine not only provides an opportunity to better understand the neurobiology of the disease and how the attack develops, but also allows for the development of new treatment options.

The pathogenesis of migraine is not fully known, but according to data from an experimental model of the disease, inflammation and oxidative stress appear to be involved [36]. In the human brain, reactive oxygen and nitrogen species (RONS) are produced during the oxidative phosphorylation (OPHOS) process in mitochondria due to intense glucose metabolism. These RONS, which are normally present at low levels, can increase with dysfunctional OPHOS as a result of the disruption of the electron transport chain (ETC). When cellular antioxidant defenses are unable to cope with increased levels of RONS, the cell falls under oxidative stress. RONS produced in this process can damage brain macromolecules, such as proteins, nucleic acids, and lipids. Alterations in neurotransmitters (such as noradrenaline, serotonin, hypocretin-1, calcitonin gene-related peptide (CGRP), and glutamate) associated with inflammation [37,38], oxidative stress, and pain are thought to play a role [37].

Nuclear factor erythroid 2-related factor 2 (Nrf2) is a transcription factor that regulates hundreds of antioxidant genes and is activated in response to oxidative stress. Keap1 (Kelch-like ECH-associated protein 1) keeps Nrf2 in the cytoplasm under normal conditions and directs it to proteasomal degradation. Under oxidative stress conditions, reactive oxygen species (ROS) cause structural alteration of Keap1, leading to the release of Nrf2 [39,40]. The released Nrf2 passes into the nucleus and protects the cell from oxidative damage by increasing the production of antioxidant enzymes. Many neurodegenerative diseases, such as Alzheimer’s, Parkinson’s, amyotrophic lateral sclerosis, Huntington’s, and multiple sclerosis, are associated with oxidative stress. It has been observed that Nrf2 is frequently activated in these diseases [41]. The Nrf2/Keap1 pathway plays a critical role in the pathogenesis of many oxidative stress-related diseases and is therefore considered as a therapeutic target.

Many studies have been conducted to understand the pathophysiology of migraine. However, a single theory has not yet been developed to explain the etiopathogenesis of the disease. Recent studies have emphasized the role of oxidative stress and related structures [42]. Although migraine is a disease closely related to oxidative stress, the Nrf2/Keap1 signaling pathway has not been sufficiently investigated in relation to the pathogenesis of this disease. This study aims to shed light on the development of new treatment approaches by revealing the relationship of this signaling pathway with migraine.

## 2. Methods

### 2.1. Ethical Approval

This study was conducted in accordance with the ethical standards outlined in the Declaration of Helsinki and was approved by the Clinical Research Ethics Committee of Gaziantep University (Decision No: 2023/278, dated 18 November 2023). All participants were thoroughly informed about the nature and purpose of this study, and written informed consent was obtained from each participant prior to enrollment.

### 2.2. Participants

In this study, 41 patients between the ages of 18 and 65 years with migraine disease who applied to Gaziantep University Faculty of Medicine Neurology Clinic were included, and 40 healthy volunteers were included as a healthy control group considering age and gender compatibility. All migraine patients were newly diagnosed and had not received any treatment. Individuals with moderate/severe mental retardation; history of major head trauma; malnutrition; pregnancy; diabetes (fasting glucose ≥ 120 mg/dL); hypertension (BP ≥ 140/90 mmHg); chronic kidney or liver disease; cancer; thyroid disorders; alcohol/substance abuse; chronic neurological diseases (e.g., epilepsy, Parkinson’s, Alzheimer’s, Huntington’s, Wilson’s disease, or prior stroke); morbid obesity; infections; or use of glucocorticoids, oral contraceptives, or antioxidants (e.g., vitamins C/E or *N*-acetylcysteine) were excluded from both groups.

### 2.3. Measurement of Parameters

Before blood collection, the patient and healthy control group were informed and their consent was obtained. An amount of 10 cc of blood was collected in anticoagulant-free tubes to examine the parameters in this study. Blood samples were kept at room temperature for 15 min. Then, they were centrifuged at 4000× *g* for 5 min and the serum samples obtained were stored in Eppendorf tubes at −80 °C until analyzed.

Plasma total antioxidant status (TAS) and total oxidant status (TOS) were measured by the fully automated colorimetric assay method developed by Erel [43,44]. In this method, the Fe^2+^-o-dianisidine complex produces the OH radical as a result of a Fenton-type reaction with hydrogen peroxide. These strong reactive oxygen species react with the colorless o-dianisidine molecule to form yellow–brown dianisidyl radicals. These radicals undergo further oxidation reactions and produce a color change. The antioxidants in the samples suppress these oxidation processes and inhibit color formation; this change is measured spectrophotometrically with automated analyzers. According to the method, thiol groups account for 52.9% and uric acid for 33.1% of the total antioxidant capacity in healthy individuals. Furthermore, oxidants convert the Fe^2+^-o-dianisidine complex into ferric ions and glycerol accelerates this reaction. Ferric ions form compounds colored xylenol orange in acidic media, and this color change is correlated with the amount of oxidants and measured spectrophotometrically. In this method, total oxidant status mostly reflects the hydrogen peroxide and lipid hydroperoxide levels. The ratio between TOS and TAS determines the oxidative stress index (OSI). The TAS and TOS values were expressed in μmol/L and OSI in an arbitrary unit.

GSK3β (Cat. No E3196Hu), Keap1 (Cat. No E5534Hu), Nrf2 (Cat. No E3244Hu), oxLDL (Cat. No E0544Hu), and Sestrin1 (Ca. No E3437Hu) were measured by measured using competitive enzyme-linked immunosorbent assay (ELISA) (BT LAB, Jiaxing Korain Biotech Co., Ltd., Jiaxing, Zhejiang, China 314021). The color intensity formed by the ELISA method was measured with an ELISA reader (Biotek ELx800, BioTek Instruments. Inc, Winooski, VT, USA). The GSK3β and Keap1 levels were expressed in ng/L, and the Nrf2, oxLDL, and Sestrin1 levels were expressed in ng/mL.

### 2.4. Statistical Analysis

The Kolmogorov–Smirnov test was used to check whether continuous variables conformed to a normal distribution. Student’s *t*-test was used to examine the differences between pairs for normally distributed data. For non-normally distributed data, the Mann–Whitney U test was used for comparisons between pairs. The Chi-square test was used to compare categorical variables and the correlation coefficient was calculated to determine the strength of the relationships between numerical variables. The data are presented as means ± standard deviation (SD). Receiver optical characteristic (ROC) curve analysis was performed to determine the optimum cut-off value of Nrf2/Keap1 to predict the presence of oxidative stress. All statistical analyses were performed using SPSS 27.0 (IBM Corp., Released 2019, Armond, NY, USA) and a value of *p* < 0.05 was considered statistically significant.

## 3. Results

Our statistical analyses showed that there were significant differences in biochemical parameters between migraine patients and the control group (Table 1). The GSK3B, Keap1, oxLDL, TOS, and OSI levels were significantly higher in migraine patients compared with the control group (*p* < 0.001). In contrast, the Nrf2, Sestrin1, and TAS levels were significantly lower in migraine patients compared with the control group (*p* < 0.001). In addition, comparisons between the patient and control groups found no statistically significant difference in terms of age and gender. These results suggest that there were differences in the regulation of oxidative stress and related biochemical mechanisms in migraine patients and suggest that these biomarkers may have been related to the pathophysiology of migraine.

**Figure 1:**
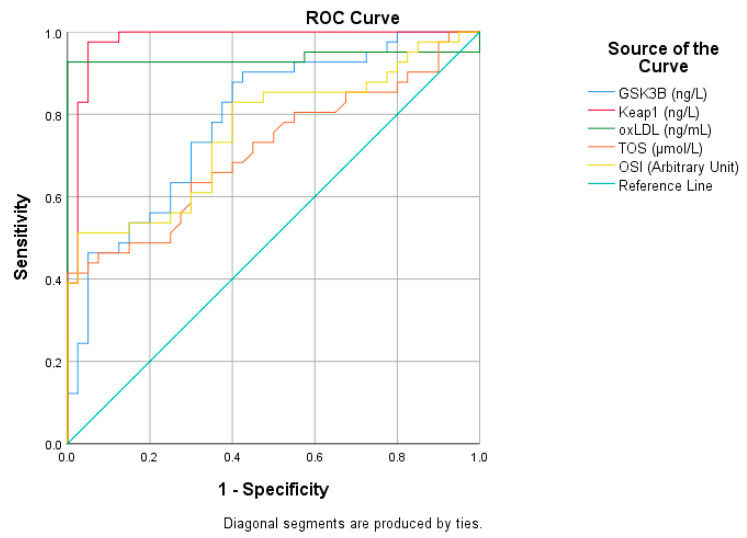
ROC analysis of ascending variables in migraine patients.

ROC curve analysis evaluates the diagnostic performance of biomarkers (Figure 1 and Table 2). According to the ROC analysis, Keap1 (red curve) was the parameter with the best diagnostic performance in differentiating migraine patients from the control group. The Keap1 curve was positioned quite far from the baseline and followed an upward trend to the left, providing high sensitivity and specificity. This suggests that Keap1 may be a strong biomarker in the diagnosis of migraine. OxLDL (green curve) also showed a remarkable performance. The similarity between Keap1 and oxLDL curves supports that both parameters are among the biomarkers that can be used in the diagnosis of migraine.

In addition, GSK3B (blue curve), TOS (orange curve), and OSI (yellow curve) were also found to have diagnostic value by significantly moving away from the baseline. However, these parameters were not as strong as Keap1 and oxLDL in terms of sensitivity and specificity. In particular, oxLDL performs quite close to Keap1 when the distance of the curve from the baseline and its overall slope are taken into account. These findings suggest that oxLDL and Keap1 stand out as biomarkers that may play an important role in the diagnosis of migraine.

**Figure 2:**
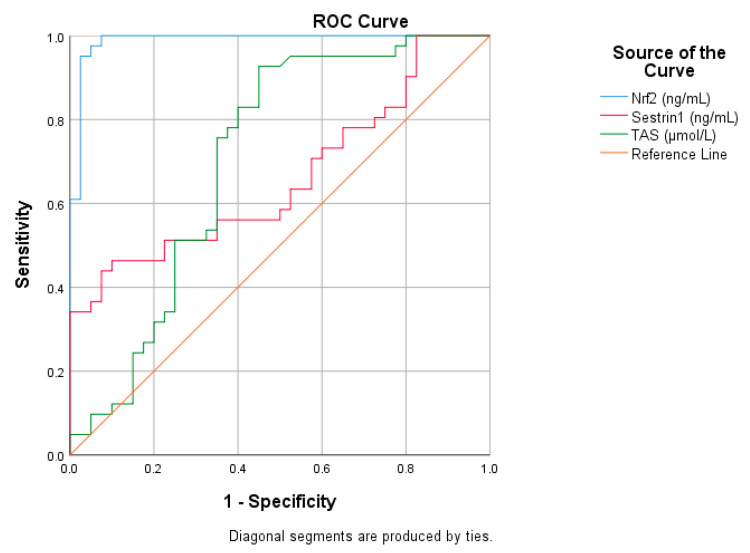
ROC analysis of declining variables in migraine patients.

ROC curve analysis evaluates the diagnostic performance of biomarkers. According to the ROC analysis, Nrf2 (blue curve) was the parameter with the best diagnostic performance in differentiating migraine patients from the control group. According to the ROC curves, the biomarker Nrf2 (ng/mL) had the highest diagnostic power. The blue curve runs very close to the upper left corner, indicating that the location and specificity of Nrf2 are quite high. This demonstrated high sensitivity and specificity of Nrf2 as a biomarker. TAS (green curve) showed a moderate performance. The green curve offers a diagnostic power as high as that of Nrf2, albeit significantly off the baseline. The biomarker Sestrin1 (red curve) showed a curve closer to the baseline and a lower diagnostic power. The red curve is considered to have low specificity, particularly as it exhibits a linear increase to the right of the graph. In general, the further away from the baseline in the ROC curve, the higher the diagnostic power. As a result of this analysis, Nrf2 stands out as the most powerful biomarker, TAS performs at an intermediate level and Sestrin1 has a more limited efficacy (Figure 2 and Table 3).

**Figure 3:**
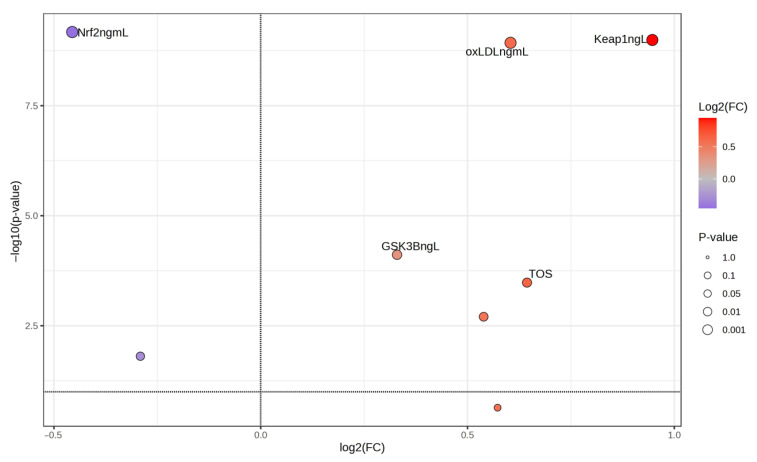
Changing parameters in migraine patients.

Figure 3 shows the change in the parameters in migraine patients compared with the control group using MetaboAnalyst 6.0 program. According to this analysis, the Keap1 and oxLDL parameters increased the most. The most decreasing value was the Nrf2 parameter (the values on the right and in red in the figure are decreasing values, while the values on the left and in blue are increasing values).

**Figure 4:**
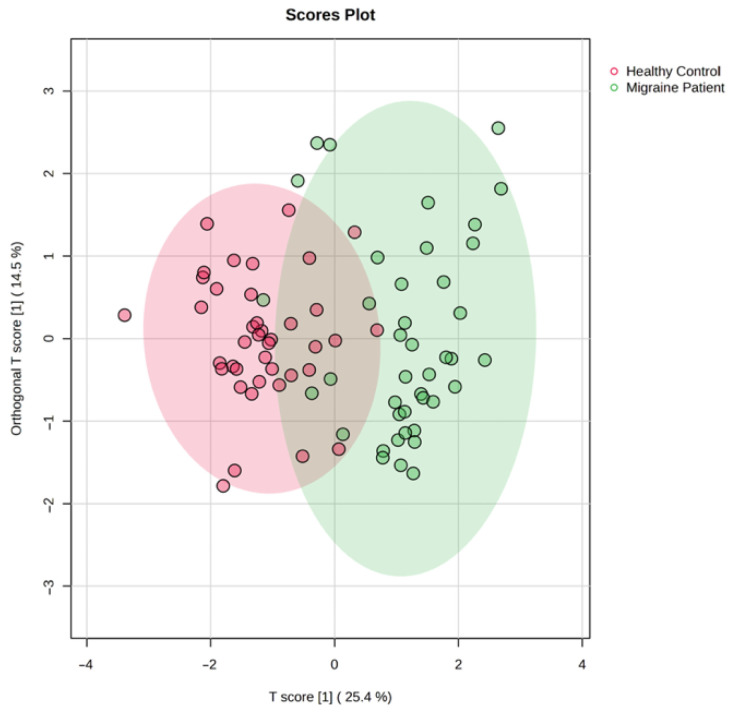
Orthogonal partial least square discriminant analysis (OPLS-DA).

Univariate and multivariate analyses were used to explore metabolites that were significantly altered between migraine patients and healthy control. An overview of the datasets using OPLS-DA multivariate analysis revealed a clear clustering and separation between the migraine patients and healthy control group, reflecting significant metabolic changes in migraine disease compared with the control group (Figure 4). T score [1] (25.4%) is the first predictor component (t[1]) in OPLS-DA, and Orthogonal T score [1] (14.5%) represents the variance in X that is not associated with the class.

**Figure 5:**
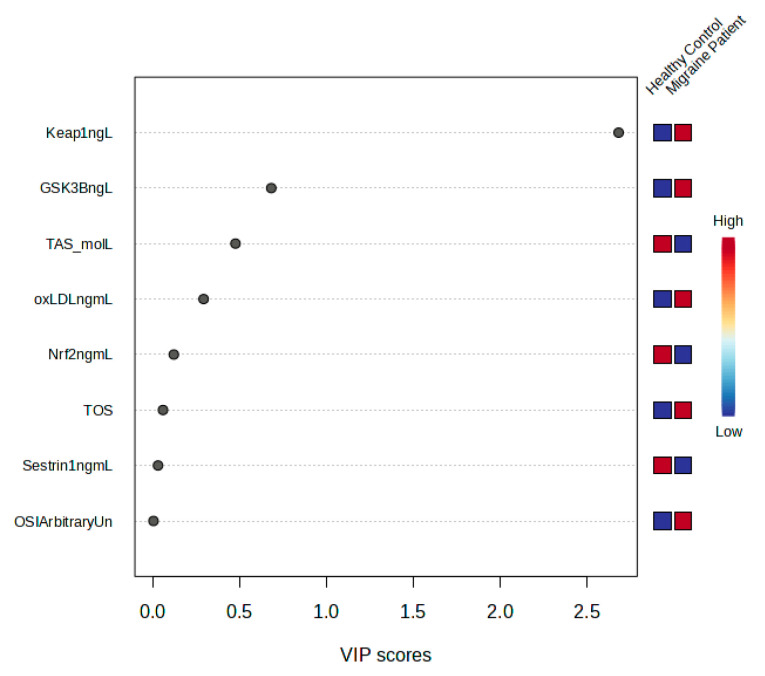
VIP Score.

A loading plot was created to identify the most important metabolites responsible for the class distinction evident in the OPLS-DA scores plot (Figure 5). Compared with the control, the migraine group exhibited higher levels of Keap1 and oxLDL, as shown in the loading plot in Figure 5. Trans Nrf2 was decreased in the migraine group compared with the control group.

Hierarchical clustering (HAC) and heat map analysis of metabolites significantly altered between the migraine group and healthy controls.

**Figure 6:**
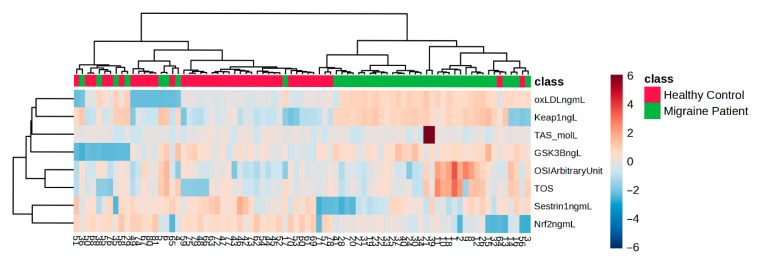
Correlation analysis of migraine and control groups.

Figure 6 shows that Keap1 and oxLDL increased, while Nrf2 and Sestrin decreased between the migraine and control groups.

## 4. Discussion

The findings of this study revealed that the Keap1 levels associated with the Nrf2 signaling pathway were significantly higher in migraine patients compared with the control group. This increase in Keap1 indicates that oxidative stress mechanisms may play an important role in migraine pathogenesis. In addition, this study found that the plasma Keap1, oxLDL, Nrf2, GSK3B, Sestrin, TOS (total oxidant level), TAS (total antioxidant level) levels, and OSI (oxidative stress index) values of migraine patients were statistically significantly different compared with the control group. These results show that oxidative stress is significantly increased in migraine patients and that, during this process, there was an increase in the Keap1, oxLDL, and GSK3B levels, whereas there was a decrease in the Nrf2, Sestrin, and TAS levels. In particular, the increase in OSI values confirms that oxidative load created a significant imbalance in migraine patients. These findings support the idea that antioxidant defense mechanisms, as well as oxidative stress, play a role in migraine pathophysiology and that regulatory elements of the Nrf2 signaling pathway should be evaluated as potential targets in migraine biology.

Migraine is one of the most common neurological diseases characterized by recurrent headache attacks that significantly affect quality of life [45]. It is defined as a neurovascular disease that includes spreading cortical depression, neurogenic inflammation, and dysfunctions in cranial vascular contractility [42]. Harmful free radicals formed during metabolic and physiological processes are normally neutralized by enzymatic and non-enzymatic antioxidant systems. However, this balance may be disrupted and oxidative stress may occur as a result of increased free radical production or inadequacy in antioxidant defense mechanisms [46].

Recent scientific studies have shown that inflammation and oxidative stress are among the important mechanisms in the triggering of migraine [47]. In particular, the release of vasoactive proinflammatory factors is thought to be the main cause of inflammation in the central nervous system, and this process is defined as neuroinflammation [48]. However, oxidative stress also appears to play a role in the pathogenesis of migraine. Oxidative stress occurs as a result of an increase in reactive oxygen species (ROS) and inadequate antioxidant defense mechanisms; this leads to damage in important biomolecules such as proteins, lipids, and DNA [42]. Similarly, the majority of studies have shown a decrease in TAS serum levels and an increase in TOS and OSI in patients with migraine [49]. The results of our current study are consistent with this study in terms of TAS, TOS, and OSI [49].

Nrf2 directly regulates the balance of reactive oxygen species (ROS) and reactive nitrogen species (RNS) by modulating antioxidant defense systems through various mechanisms. Thus, it plays a critical role in maintaining cellular homeostasis [50]. Activated Nrf2 affects oxidant physiology and pathology by increasing the expression of various enzymes and signaling proteins related to drug metabolism, antioxidant defense mechanisms, and oxidant signaling. Nrf2 plays a role in the control of autophagy, inflammasome signaling, endoplasmic reticulum stress response (UPR), apoptosis, mitochondrial biogenesis, and stem cell functions by regulating oxidant levels and signaling mechanisms. In addition, natural or pharmacological activation of Nrf2 has multifaceted protective effects against toxicity and chronic diseases, and this feature offers important opportunities for drug development processes [50].

The decrease in Nrf2 levels and the increase in Keap1 levels in our study are consistent with the findings reported in the current literature. It is thought that the decrease in Nrf2 occurs due to the disruption of the oxidant/antioxidant balance in the body. This situation reveals that Nrf2 plays an important role in combating oxidative stress by regulating antioxidant defense systems. Our findings are supported by the significant decrease in the levels representing total antioxidant capacity (TAS) and the significant increase in total oxidant status (TOS) levels. While the decrease in TAS levels indicates that cellular antioxidant defense mechanisms are inadequate and the oxidative load increases, the increase in TOS levels reveals that systemic oxidative stress has become apparent. Accordingly, the Keap1-Nrf2 signaling pathway is thought to play a critical role in the pathophysiological processes associated with oxidant/antioxidant imbalance.

This finding suggests that Nrf2 is not regulated in the same way in all diseases and that a disease-specific redox adaptation may exist. Indeed, while Nrf2 activation increases in some conditions associated with oxidative stress, such as neurodegenerative diseases, COPD, and aging [50], suppression of this pathway can be observed in diseases characterized by recurrent, acute, and predominantly neuroinflammatory stress, such as migraine. The significant decrease in Nrf2 found in migraineurs in our study suggests that the antioxidant defense system is suppressed and that this response may have become depleted or dysfunctional over time. This suggests that a phenomenon described in the literature as a “disease-specific redox response” (disease-specific redox adaptation) may also apply to the context of migraine [50]. Therefore, the low levels of Nrf2 in migraineurs suggests that this pathway is suppressed in pathophysiological processes and may be a targetable mechanism for both diagnostic and therapeutic purposes.

The relationship between lipid levels and migraine has recently become an interesting area of research [51,52]. In a population-based study conducted in the Netherlands, the total cholesterol level and total cholesterol/HDL-C ratio were shown to be associated with migraine with aura [53]. Similarly, in a clinical study in Austria, the total cholesterol, LDL, and oxidized LDL levels were found to be significantly higher in migraine patients compared with the control group [52]. In our study, similar to previous studies, oxLDL levels were found to be significantly higher in migraine patients compared with the control group. This suggests that oxidative stress is associated with increased lipid peroxidation in migraineurs. oxLDL is the oxidation product of LDL under oxidative stress conditions and can trigger inflammatory processes in the vascular endothelium, causing vascular dysfunction. Increased oxLDL levels may be associated with vascular inflammation and trigeminal nerve activation during migraine attacks. It may also be considered a reflection of neuroinflammation that plays a role in migraine pathophysiology.

Dysregulation of GSK-3β plays an important role in the pathogenesis of various diseases, including cancers [54], diabetes [55], and neuroinflammatory and neurodegenerative diseases [56,57]. Neuroinflammation is defined as a regulated biological response that develops in response to infectious agents or tissue damage and is characterized by increased glial activation, increased proinflammatory cytokine concentrations, increased blood–brain barrier (BBB) permeability, and leukocyte infiltration [58]. In our study, a significant increase in GSK3B levels was observed in migraine patients. GSK3B is a serine/threonine kinase that plays a critical role in various cellular processes, especially energy metabolism, cellular growth, and apoptosis. Increased GSK3B levels may be associated with inflammatory processes triggered by oxidative stress. In addition, GSK3B activation may be associated with increased neuroinflammation and pain sensitivity during migraine attacks. This finding suggests that GSK3B may be a potential target in migraine pathogenesis.

Sestrins are evolutionarily conserved, stress-induced proteins that are activated against various cellular threats such as oxidative stress, hypoxia, and genotoxic stress [59]. Sestrins have an important effect in balancing reactive oxygen species (ROS) and reactive nitrogen species (RNS). While directly regulating the antioxidant defense, it supports cellular homeostasis by protecting mitochondrial functions and increasing mitophagy [60]. In addition, Sestrin2 has been shown to play a protective role by regulating oxidative stress and autophagy in aging-related disorders such as neurodegenerative diseases [61]. Sestrin1 (PA26) is a stress-induced protein and regulates cellular protection mechanisms especially in harsh conditions such as oxidative stress, hypoxia, and genotoxic stress [62]. One of the most fundamental roles of Sestrin1 is to regulate the metabolism of reactive oxygen species (ROS) and increase antioxidant defense at the cellular level [62]. This feature plays an important role in preventing DNA damage and slowing down cellular aging. Sestrin1 also supports cellular processes such as autophagy, regulating energy balance, and increasing cell survival [62]. With these features, Sestrins stand out as potential therapeutic targets for neurodegenerative diseases such as Alzheimer’s, Parkinson’s, and Huntington’s disease. It was determined that Sestrin1 levels were significantly reduced in migraine patients compared with the control group. Sestrins are proteins that play an important role in the oxidative stress response and protect against oxidative damage by regulating cellular redox balance. The decrease in Sestrin1 levels supports the inadequacy of antioxidant defense mechanisms and increased oxidative stress load in migraineurs. This may lead to neuronal cells becoming more susceptible to oxidative damage during migraine attacks. The decrease in Sestrin1 levels may indicate the inadequacy of neuroprotective mechanisms in migraineurs. This suggests that neurons become more vulnerable to oxidative damage during migraine attacks, and it may be beneficial to develop antioxidant treatments for this condition.

Our study demonstrates that oxidative stress and inflammation play critical roles in the pathogenesis of migraine. The increased Keap1 levels, decreased Nrf2 levels, and impaired TAS–TOS balance in migraine patients support the idea that oxidative stress is a central factor in this disease. In particular, irregularities in the Keap1-Nrf2 signaling pathway confirm the inadequacy of antioxidant defense mechanisms and their effects on migraine pathophysiology. In addition, increased GSK3B and decreased Sestrin1 levels indicate that neuroinflammation and cellular stress make significant contributions in individuals with migraine. The significant increase in oxLDL levels, an indicator of lipid peroxidation, underlines the relationship between migraine and vascular inflammation and endothelial dysfunction. The significant increase in OSI values showed that oxidative load created a significant imbalance in patients with migraine. These findings reveal that oxidative stress, inflammation, and antioxidant defense mechanisms work together in migraine biology.

The findings suggest that approaches aimed at reducing oxidative stress may be potentially effective in the treatment of migraine. Pharmacological agents targeting the Keap1-Nrf2 signaling pathway may offer important therapeutic strategies for regulating oxidative stress and improving antioxidant defense mechanisms. In addition, GSK3B inhibitors and Sestrin1 activators may be investigated to control migraine-associated inflammation and neurovascular dysfunction. Reducing oxLDL levels or preventing lipid peroxidation may also contribute to reducing the frequency and severity of migraine attacks. In future studies, the clinical efficacy of treatment approaches targeting oxidative stress and inflammation should be evaluated. In addition, further research is recommended for the use of these biomarkers in the early diagnosis of the disease and the development of individualized treatment strategies.

## 5. Conclusions

Our study findings revealed that Keap1, oxidized LDL (oxLDL), Glycogen Synthase Kinase-3 Beta (GSK3B), total oxidant level (TOS), and oxidative stress index (OSI) levels were significantly higher in migraine patients compared to controls. Conversely, Nrf2, Sestrin1, and total antioxidant (TAS) levels were significantly lower in migraine patients.

The decrease in Nrf2 levels and the increase in Keap1 levels confirm that the antioxidant defense system is inadequate in migraine patients, leading to increased oxidative stress. The significant increase in OSI values, a key indicator of oxidative stress, clearly indicates that the oxidative/antioxidant balance is disrupted in these patients. Additionally, elevated oxLDL levels suggest increased lipid peroxidation and that migraine may be associated with vascular inflammation. The increase in GSK3B and the decrease in Sestrin1 indicate that neuroinflammation and cellular stress are important mechanisms contributing to migraine pathophysiology.

In light of these findings, the Nrf2/Keap1 signaling pathway may be considered a potential therapeutic target for migraine treatment. The development of new treatment strategies that target oxidative stress and strengthen antioxidant defense mechanisms may be beneficial in migraine management. It is recommended that these biomarkers be examined in more detail in future studies to facilitate early diagnosis and develop personalized treatment approaches.

## Figures and Tables

**Table 1:** Statistical analysis of ELISA results.

	Migraine Group			Control Group			*p* ^a^
	Min	Max	Mean ± SD	Min	Max	Mean ± SD	
GSK-3β (ng/L)	209.83	385.53	285.87 ± 39.39	174.53	327.78	242.41 ± 37.27	0.001 **
Keap1 (ng/L)	353.79	571.10	480.65 ± 42.24	153.90	493.54	257.3 ± 81.57	0.001 **
Nrf2 (ng/mL)	22.04	34.67	28.92 ± 3.09	30.22	46.10	39.22 ± 3.31	0.001 **
oxLDL (ng/mL)	42.65	86.90	72.74 ± 8.64	44.17	65.31	52.22 ± 4.52	0.001 **
Sestrin1 (ng/mL)	7.37	16.91	12.54 ± 3.27	10.01	21.23	14.69 ± 2.41	0.012 *
TOS (μmol/L)	5.77	28.53	14.06 ± 5.74	5.13	14.80	10.16 ± 2.4	0.001 **
TAS (μmol/L)	675.10	1076.50	877.54 ± 87.86	719.80	1286.00	973.4 ± 140.94	0.001 **
OSI (Arbitrary Unit)	0.57	3.62	1.62 ± 0.69	0.51	1.79	1.07 ± 0.31	0.001 **

^a^ Mann–Whitney U test, * *p* < 0.05, ** *p* < 0.001, Migraine Group (n = 41), Control Group (n = 40).

**Table 2:** ROC analysis of increasing parameters in migraine patients.

Risk Factor	AUC (%95)	Cutt Off	*p*	Sensitivity (%)	Specificity (%)
GSK3B (ng/L)	0.788 (0.690–0.886)	263.14	0.001	70	70,000
Keap1 (ng/L)	0.978 (0.947–1)	393.21	0.001	98	95
oxLDL (ng/mL)	0.937 (0.867–1)	65.14	0.001	93	100
TOS (μmol/L)	0.714 (0.601–0.827)	10.8	0.001	66	65
OSI (Arbitrary Unit)	0.759 (0.654–0.865)	1.12	0.001	73	65

**Table 3:** ROC analysis of increasing parameters in migraine patients.

Risk Factor	AUC (%95)	Cutt Off	*p*	Sensitivity (%)	Specificity (%)
Nrf2 (ng/mL)	0.988 (0.968–1)	33.36	0.001	95	98
Sestrin1 (ng/mL)	0.662 (0.542–0.781)	13.87	0.012	56	65
TAS (μmol/L)	0.706 (0.558–0.825)	928.65	0.001	75	65

## Data Availability

The datasets used during the current study are available from the corresponding authors upon request.
